# Kinetics of Glucoregulatory Peptide Hormones during Hemodialysis with Cellulose Triacetate and Polysulfone Dialyzers in Patients with Diabetes and End-Stage Kidney Disease

**DOI:** 10.3390/ijms241310604

**Published:** 2023-06-25

**Authors:** Nobuteru Takao, Takashi Maruyama, Hiroki Kobayashi, Maki Kitai, Yoshinori Yoshida, Hiroyuki Takashima, Masanori Abe

**Affiliations:** Division of Nephrology, Hypertension and Endocrinology, Department of Medicine, Nihon University School of Medicine, Itabashi City, Tokyo 173-8610, Japanmaruyama.takashi@nihon-u.ac.jp (T.M.); kobayashi.hiroki@nihon-u.ac.jp (H.K.);

**Keywords:** cellulose triacetate, glucagon, hemodialysis, insulin, polysulfone, super high-flux dialyzer

## Abstract

The mechanisms behind reported decreases in plasma insulin and glucagon during hemodialysis (HD) are not clear. Here, we investigated these mechanisms during HD treatment and the characteristics of insulin and glucagon removal when using two super high-flux membranes. In an experimental study, clearance, adsorption rates, and reduction rates of insulin and glucagon were investigated when using cellulose triacetate (CTA) and polysulfone (PS) membranes in a closed circuit using bovine blood. In a clinical study, 20 diabetes patients with end-stage kidney disease who were stable on HD were randomly selected for two HD sessions with two different membranes. At 1 h after the initiation of HD, insulin and glucagon clearance were measured, and the reduction rates were also investigated. In the experimental study, the PS membrane showed significantly higher clearance, adsorption rates, and reduction rates of insulin and glucagon compared with the CTA membrane. Although glucagon was detected in the ultrafiltration fluids in both membranes, insulin was absent in the PS membrane. In the clinical study, both membranes showed significant reductions in plasma insulin and glucagon at each time point. The PS membrane showed significantly higher insulin clearance and reduction rates compared with the CTA membrane. The two membranes showed no significant difference in glucagon clearance, but the glucagon reduction rate was significantly higher with the PS membrane. Our findings show that HD with the two super high-flux membranes used removes significant amounts of glucoregulatory peptide hormones from plasma in patients with diabetes and end-stage kidney disease, potentially affecting their glucose metabolism.

## 1. Introduction

The most common cause of end-stage kidney disease and the start of renal replacement therapy, including hemodialysis (HD), is diabetes mellitus [1]. Even in patients with diabetes undergoing HD, maintaining excellent glycemic control is crucial to prevent micro- and macrovascular problems and infection [2]. However, there are variations in the dynamics of many hormones between HD patients. Moreover, it has been reported that glycemic disarray is often observed in patients with diabetes undergoing HD [3]. Glycemic disarray, which frequently occurs in HD patients, especially during HD sessions, includes HD-induced hypoglycemia and HD-associated hyperglycemia and may be related to the dynamics of these hormones. Thus, it is crucial to look at the hormonal dynamics in patients undergoing HD, particularly those of insulin and glucagon. Because numerous biological reactions can occur when artificial materials and blood components come into contact during extracorporeal circulation, changes in plasma insulin and glucagon levels may vary depending on the type of membrane used. The insulin dosage must be changed depending on the type of membrane used for HD therapy or on the days that HD treatment is given if substantial discrepancies between plasma insulin and glucagon levels are observed in HD performed using different membranes.

It has been observed that throughout the HD session, patients receiving human insulin saw a decrease in plasma insulin levels and that the clearance rate varied depending on the type of high-flux membrane used [4,5,6]. Recently, we showed improved mortality in patients on HD with the use of super high-flux membranes [7]. However, no studies have looked into how patients taking insulin analogs change their plasma insulin dynamics. In addition, the dynamics of plasma glucagon levels during HD therapy with varying membrane types have not been documented. The current study was designed to better understand the removal of insulin and glucagon during HD treatment, as well as the properties of removal using two super high-flux membranes.

## 2. Results

### 2.1. Experimental Study

**Figure 1:**
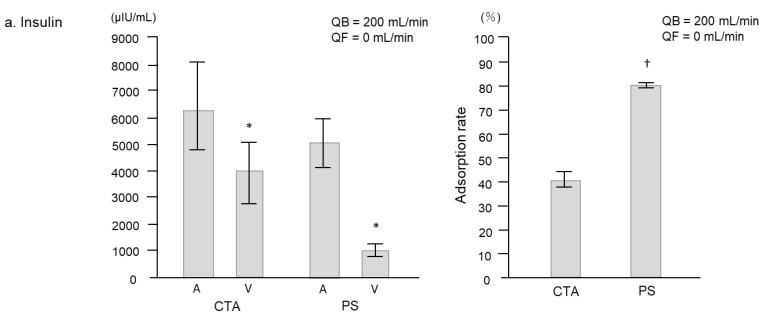

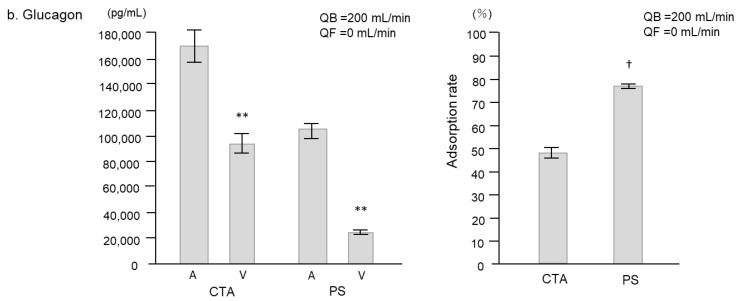
Concentrations and adsorption rates of insulin and glucagon at 1 min after the start of circulation without ultrafiltration in the CTA and PS membranes. (**a**) Insulin concentrations at A and V sites and adsorption rate in CTA and PS membranes. (**b**) Glucagon concentrations at A and V sites and adsorption rate in CTA and PS membranes. Data are shown as mean ± standard error. * *p* < 0.05 and ** *p* < 0.01 vs. A. † *p* < 0.01 vs. CTA. A, Arterial site; CTA, cellulose-triacetate; PS, polysulfone; QB, blood flow rate; QF, ultrafiltration rate; V, venous site.

As shown in Figure 1a, the concentration of insulin at 1 min after the start of circulation without ultrafiltration significantly decreased with each membrane type: from 6433 ± 1707 μIU/mL at arterial (A) site to 3933 ± 1335 μIU/mL at venous (V) site with the cellulose triacetate (CTA) membrane (*p* = 0.03) and from 4913 ± 1142 μIU/mL to 995 ± 289 μIU/mL with the polysulfone (PS) membrane (*p* = 0.03). There was a significant difference in the adsorption rate of insulin between the two membranes (40.4 ± 6.3% vs. 80.5 ± 1.4%, respectively; *p* = 0.003). Similarly, as shown in Figure 1b, the concentration of glucagon at 1 min after the start of circulation without ultrafiltration significantly decreased with each membrane type: from 177,333 ± 7513 pg/mL at site A to 90,666 ± 10,671 pg/mL at site V with the CTA membrane (*p* = 0.004) and from 114,667 ± 3332 pg/mL to 29,166 ± 937 pg/mL with the PS membrane (*p* = 0.001). There was a significant difference in the adsorption rate of glucagon between the membranes (49.1 ± 4.1% and 74.6 ± 0.6%, respectively; *p* = 0.004).

**Figure 2:**
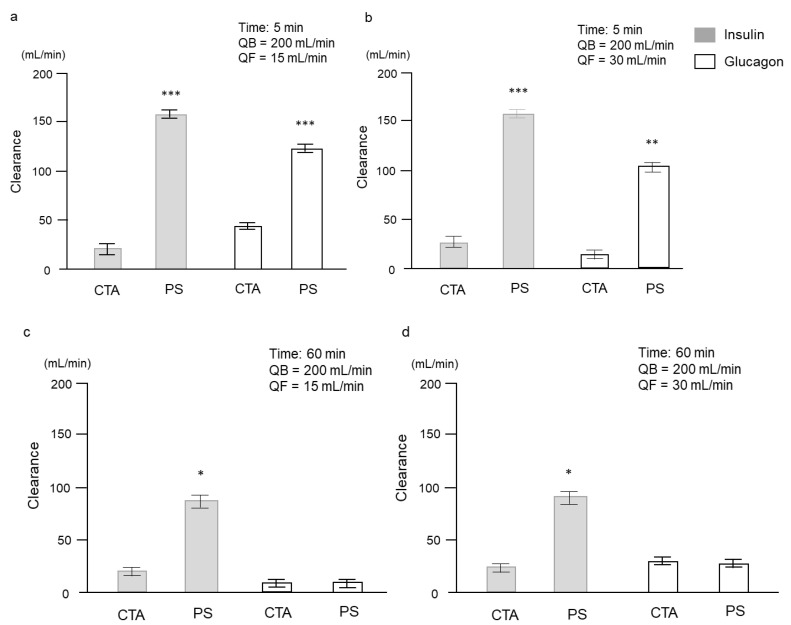
Comparison of insulin and glucagon clearance with each dialyzer membrane (**a**) at 5 min after circulation with QF of 15 mL/min, (**b**) at 5 min after circulation with QF of 30 mL/min, (**c**) at 60 min after circulation with QF of 15 mL/min, and (**d**) at 60 min after circulation with QF of 30 mL/min. Data are shown as mean ± standard error. * *p* < 0.01, ** *p* < 0.001, and *** *p* < 0.0001 vs. CTA. CTA, cellulose-triacetate; PS, polysulfone; QB, blood flow rate; QF, ultrafiltration rate.

As shown in Figure 2, insulin clearance was significantly higher with the PS membrane than with the CTA membrane. No insulin was detected in the ultrafiltrate fluid with the PS membrane when the ultrafiltration rate (QF) was 15 and 30 mL/min (Table 1). Although glucagon clearance at 5 min was significantly higher with the PS membrane than with the CTA membrane, there was no significant difference at 60 min regardless of the value of the QF. Glucagon was detected in the ultrafiltrate fluids with each membrane when the QF was 15 and 30 mL/min (Table 1).

**Figure 3:**
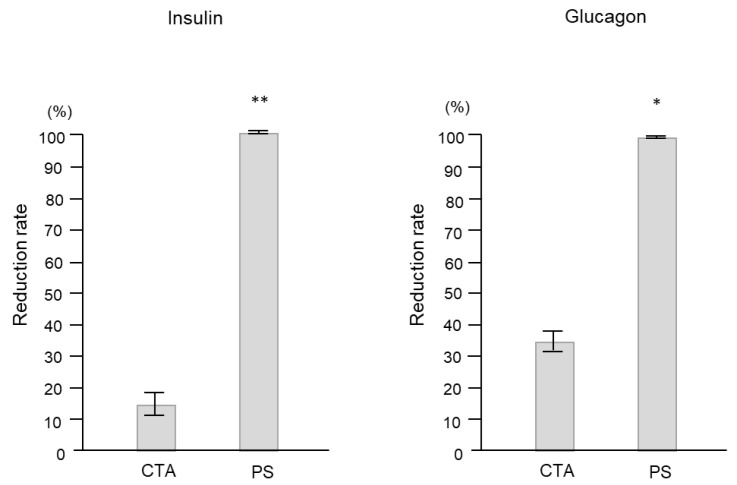
Comparison of insulin and glucagon reduction rates according to the dialyzer membrane used. Data are shown as mean ± standard error. * *p* < 0.001 and ** *p* < 0.0001 vs. CTA. CTA, Cellulose-triacetate; PS, polysulfone.

As shown in Figure 3, the reduction rates of insulin and glucagon were significantly higher with the PS membrane than with the CTA membrane (insulin: 99.2 ± 0.1% vs. 15.1 ± 4.9%, *p* < 0.0001; glucagon: 98.2 ± 0.2% vs. 34.3 ± 0.6%, *p* = 0.0002).

### 2.2. Clinical Study

**Figure 4:**
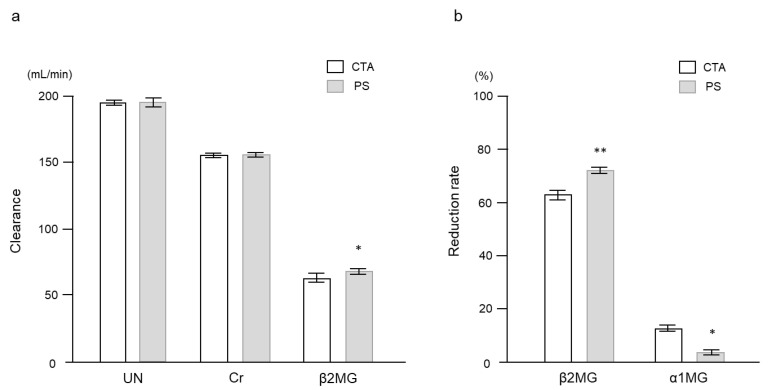
Comparison of solute clearance and reduction rates between the two dialyzer membranes. (**a**) Clearance. (**b**) Reduction rate. Data are shown as mean ± standard error. * *p* < 0.001 and ** *p* < 0.0001 vs. CTA. α1MG, α_1_-microglobulin; β2MG, β_2_-microglobulin; Cr, creatinine; CTA, cellulose-triacetate; PS, polysulfone; UN, urea nitrogen.

Table 2 shows the baseline characteristics of the patients. Mean age was 70.8 ± 1.1 years, and the median duration of the HD treatments was 46 (37–69) months. All patients were anuric. Mean hemoglobin A1c and GA levels were 6.0 ± 0.1% and 20.3 ± 0.3%, respectively. Six patients (30.0%) received insulin injection therapy, and twelve received oral antidiabetic agents. There was no significant difference in serum urea nitrogen (UN) or creatinine (Cr) clearance between the two membranes (Figure 4). However, the clearance and reduction rate of β_2_-microglobulin (β2MG) was higher with the PS membrane. On the other hand, the reduction rate of α_1_-microglobulin (α1MG) was higher with the CTA membrane.

**Figure 5:**
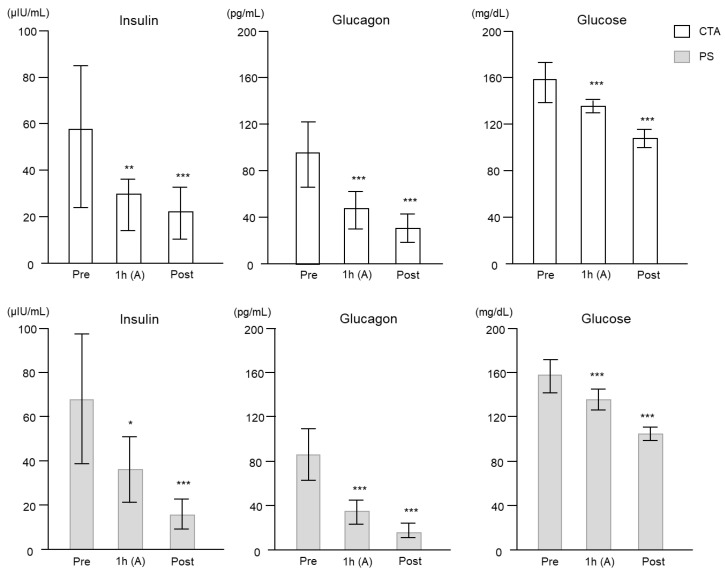
Changes in plasma insulin, glucagon, and glucose levels between the arterial (A) site samples during hemodialysis at each time point for each dialyzer membrane used. Data are shown as mean ± standard error. * *p* < 0.01, ** *p* < 0.001, and *** *p* < 0.0001 vs. Pre.

Plasma insulin, glucagon, and glucose values during HD (Figure 5) significantly decreased at each time point with each type of membrane used. Post-HD glucose and insulin levels did not differ according to the membrane used.

**Figure 6:**
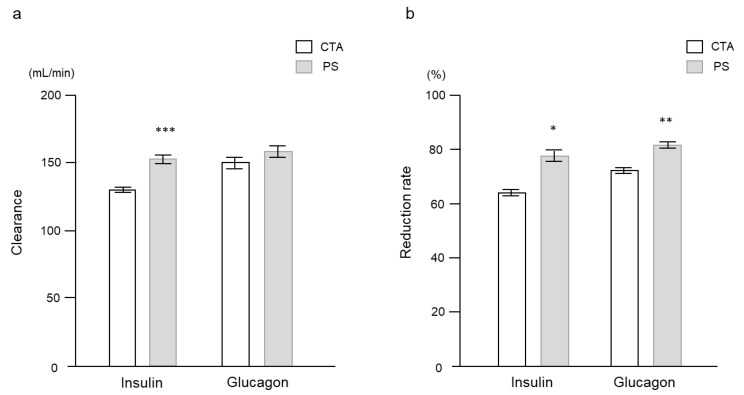
(**a**) Comparison of insulin and glucagon clearance at 1 h after initiating hemodialysis, and (**b**) reduction rates between the two dialyzer membranes. Data are shown as mean ± standard error. * *p* < 0.05, ** *p* < 0.01, and *** *p* < 0.0001 vs. CTA. CTA, Cellulose-triacetate; PS, polysulfone.

There was a significant difference in insulin clearance (Figure 6a) but not in glucagon clearance (Figure 6b) between the two membranes. The reduction rates of insulin and glucagon were significantly higher with the PS membrane than with the CTA membrane (Figure 6b).

## 3. Discussion

This is the first study in which plasma glucagon was removed by HD, and its levels decreased during HD sessions. Adsorption and convection served as the mechanisms by which HD removed glucagon. Contrarily, during HD with the PS membrane in the experimental investigation, insulin was not found in the ultrafiltrate fluid. As a result, it was hypothesized that during HD with the PS membrane, plasma insulin was eliminated by adsorption rather than convection. Furthermore, the use of various dialyzer membrane types resulted in varying degrees of insulin and glucagon adsorption. In the experimental study, plasma glucagon and insulin levels were cleared more quickly from the PS membrane than from the CTA membrane. As a result, the reduction rates of insulin and glucagon were higher in the PS membrane than in the CTA membrane in the clinical study.

Dialyzers in Japan are divided into five categories based on how well they clear β2MG at a blood flow rate of 200 mL/min and a dialysate flow rate of 500 mL/min [8,9]: type I is categorized as a low-flux membrane dialyzer (10 mL/min clearance), types II and III are categorized as high-flux membrane dialyzers (10–30 mL/min and 30–50 mL/min clearance, respectively), and types IV and V are categorized as super high-flux membrane dialyzers (50–70 mL/min and ≥70 mL/min clearance, respectively). Since 2008, super high-flux membrane dialyzers have been used to treat more than 90% of Japanese HD patients [10]. The CTA and PS membranes used in this study were classified as type IV super high-flux membranes. Therefore, β2MG clearance was achieved above 50 mL/min in both membranes. Furthermore, because the pore size of the PS membrane was smaller than that of the CTA membrane, the α1MG reduction rate of the PS membrane was lower than that of the CTA membrane. However, it is also necessary to consider the possibility that the blood-glucose-regulating hormone is adsorbed within the dialyzer. Numerous studies have been conducted on the adsorption of proteins on biological surfaces. This process is extremely complex, and a variety of variables, including electrostatic interactions and hydrophilicity or hydrophobicity, can have an impact [11,12,13]. Better knowledge of this mechanism would be helpful for the research of protein transport pathways within membranes because protein adsorption to membranes may alter the pore size and charge characteristics of the membrane [14,15,16].

The clearance of insulin varied with different types of membranes, and it has been reported that the plasma insulin level after passing through the dialyzer was lower than the level before [17]. However, a previous study found the dynamics in patients who were treated with human insulin [5]. Conversely, insulin analogs could not be detected by the assay used in the present study. Therefore, this study confirmed the dynamics of endogenous insulin during HD treatment. Because plasma insulin has a 1% protein binding rate and a molecular weight of 6.2 kDa, it is theorized that plasma insulin may be eliminated by diffusion and/or convection mechanisms. Although the exact mechanism for insulin reduction—whether it is caused by convection or adsorption—has not yet been determined by various dialyzer membranes, it can be inferred from the current findings that it is entirely caused by adsorption. From the current findings, we can speculate that it is entirely caused by adsorption for PS membranes and by adsorption and convection for CTA membranes. Zeta potential identifies the electric charge of hollow-fiber dialysis membranes [18]. The electrostatic effect is an important factor for insulin adsorption, and a higher equilibrium level of adsorption occurs when insulin carries the opposite charge to the membrane.

Plasma glucagon levels decreased after passage through a dialyzer. As a mechanism, we demonstrated that plasma glucagon is removed by dialyzers via adsorption and filtration in the experimental study and diffusion in the clinical study. The 29-amino-acid residue peptide hormone glucagon plays crucial roles in the metabolism of proteins and amino acids [19]. As a small-middle molecule with a molecular weight of 3.5 kDa, glucagon can cross super high-flux membranes like the PS and CTA membranes. Furthermore, the isoelectric point of glucagon is approximately pH 5.8–6.0. Plasma glucagon is positively charged in a pH 7.4 context and is therefore adsorbed on negatively charged CTA and PS membranes [20]. Therefore, glucagon is removed by HD in a manner independent of plasma glucose levels, suggesting an inability to respond to HD-related hypoglycemia.

Because glucose has a very low molecular weight and rapidly crosses the membrane during HD therapy, the plasma glucose levels at the post-HD stage were mostly dictated by the glucose concentration in the dialysate, which was 100 mg/dL in the current study. Therefore, regardless of the type of super high-flux membrane, the plasma glucose levels at the post-HD stage should be comparable for both membranes. According to reports, insulin levels during an HD session depend on both the removal of insulin and the secretion of insulin from the pancreatic β-cells. These factors are, in turn, influenced by changes in plasma glucose brought on by HD and the β-cells’ capacity to react to these glucose changes [21]. Because all patients’ plasma glucose levels were higher than 100 mg/dL before HD, the plasma glucose levels in the current study steadily decreased during the HD session due to removal by diffusion into the dialysate. Therefore, it was suggested that this was because there was no need to secrete insulin from pancreatic β-cells. In addition, plasma insulin was removed by the HD procedure independent of changes in plasma glucose levels. Therefore, it was suggested that the decrease in plasma insulin levels during HD did not cause an increase in plasma glucose levels along with the reduction in plasma glucagon levels during HD. Glucoregulatory peptide hormones, which include insulin and glucagon, were removed regardless of the plasma glucose levels in the present study. In addition, it has been reported that approximately 10 to 12 g of amino acids and several grams of proteins are removed during each HD treatment [22,23]. Therefore, HD is a catabolic procedure. The PS membrane had higher plasma insulin clearance and reduction rates compared with the CTA membrane. Therefore, HD-associated hyperglycemia may occur more frequently when the PS membrane is used. As a result, it may be helpful for patients to monitor their blood glucose on the day that HD is conducted so that they can assess their own glycemic status. If hyperglycemia is identified, an additional dose of injectable insulin will be required in the following HD therapy. In addition, continuous glucose monitoring might be useful for detecting HD-associated hyperglycemia.

This study had several limitations. First, some patients received DPP-4 inhibitors. Therefore, the secretion of glucagon might be suppressed. Glycated albumin is recommended as a glycemic control in HD patients because glycated hemoglobin significantly underestimates glycemia levels in HD patients due to the presence of renal anemia, its treatment with the erythropoiesis-stimulating agent, and its reduced erythrocyte lifespan [2,3]. This participant had a mean GA level of 20.3 ± 0.3% and thus included patients with relatively stable glycemic control. Second, the effects of diet have not been considered. In this study, postprandial blood samples were taken as pre-HD samples because the patients arrived at the hospital after breakfast. Finally, all patients who were treated with insulin therapy received insulin analogs and not human insulin. Insulin analogs could not be detected by chemiluminescent enzyme immunoassay. Therefore, we could only investigate the kinetics of the endogenous insulin concentration. Further studies are required to clarify the kinetics of insulin analogs during HD sessions.

In conclusion, this study was the first to assess the clearance and reduction rates of insulin and glucagon in patients with diabetes undergoing HD. In the experimental study, we revealed that plasma glucagon was removed during HD because of absorption and convection in two super high-flux dialyzers, which were CTA and PS membranes. However, the removal mechanism of insulin by HD differed between the CTA and PS membranes. Plasma insulin was removed by adsorption and convection in the CTA membrane, while it was removed by only adsorption in the PS membrane. In the clinical study, the clearance of insulin was higher in the PS membrane compared to the CTA membrane. The reduction rates of glucagon and insulin were higher in the PS membrane than in the CTA membrane. During HD, using the two super high-flux membranes, plasma concentrations of glucose, insulin, and glucagon all decreased over time. The mechanism of removal of glucagon and insulin may differ depending on the type of super high-flux membrane used in HD, which requires further investigation. To assess HD-related hypoglycemia and HD-associated hyperglycemia in patients with diabetes undergoing HD, more research on glucagon and insulin dynamics is required.

## 4. Materials and Methods

### 4.1. Experimental Study

**Figure 7:**
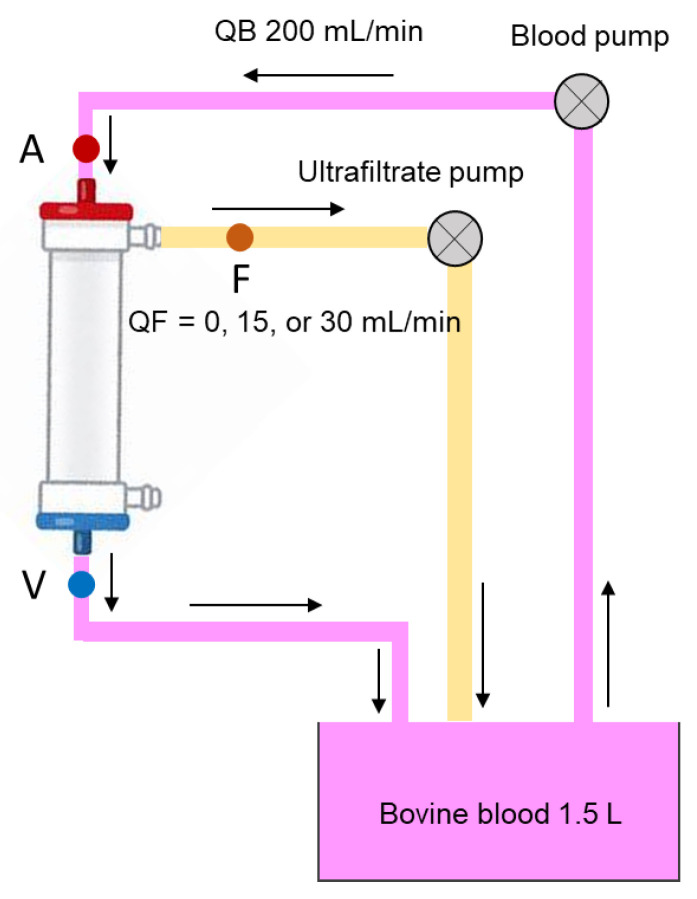
Schematic of the experimental closed circuit. A, Arterial sampling site; F, ultrafiltration fluids sampling site; QB, blood flow rate; QF, ultrafiltration rate; V, venous sampling site. Arrows indicate direction of flow.

Batches of 1500 mL of bovine blood were prepared with phosphate-buffered solution (pH 7.4), 40 units of biosynthetic human insulin, and 1 mg of genetical recombinant glucagon. From the tank, the bovine blood was pumped to the dialyzers at blood flow rate (QB) of 200 mL/min in the closed circuit (Figure 7). It was anticoagulated by injecting 10,000 units of heparin and adjusted to hematocrit (Ht) values of 30 ± 2.0% and total protein levels of 6.5 ± 0.5 g/dL. The bovine blood was maintained at 37 ± 1 °C throughout the experiments. Insulin injections comprised biosynthetic human insulin (Humulin R, Eli Lilly, Indianapolis, IN, USA), and glucagon injections comprised genetical recombinant glucagon (Glucagon G Novo Injection, Novo Nordisk Pharma, Bagsværd, Denmark). Supplementary Table S1 lists the characteristics of the two hollow-fiber dialyzers used (*n* = 4 each): the FB-150Uβeco with a CTA membrane (1.5 m^2^ surface area; Nipro, Osaka, Japan) and the APS-15SA with a PS membrane (1.5 m^2^ surface area; Asahi Kasei Medical Co., Ltd., Tokyo, Japan). A commercial polyvinylchloride tubing set (NV-Y/Z; Nikkiso, Tokyo, Japan) was used as the connecting line.

Using another roller pump, ultrafiltration across each type of membrane was induced at QFs of 0, 15, or 30 mL/min. Bovine blood was allowed to circulate for 60 min. For adsorption evaluation, blood samples were obtained from the blood tubing at the A and V sites of the dialyzer after 1 min at QF of 0 mL/min and then again at the A and V sites of the dialyzer and from the ultrafiltrate fluids (F) site after 5 min and 60 min at QFs of 15 and 30 mL/min, as described in our previous study [5].

Insulin and glucagon removal were compared according to the two membrane types using the clearance values for insulin and glucagon calculated using the following equation: K = QB × (Cart. − Cven.)/(Cart.) + QF × (Cven/Cart.). Here, K represents the clearance rate (mL/min), QB represents the blood flow (mL/min), QF represents the ultrafiltration rate (mL/min), Cart. represents the concentration at the A site of the dialyzer, and Cven. represents the concentration at the V site of the dialyzer [5]. Adsorption rate was calculated according to following equation at 1 min after the circulation without ultrafiltration: adsorption rate = (Cart. − Cven.)/Cart × 100 [5]. Reduction rate (%) was calculated according to the following equation adjusted to hematocrit (Ht) at 60 min after the circulation: {1 − Cpost/Cpre × (1 − Htpost/100)/(1 − Htpre/100) × Htpre/Htpost} × 100. Here, Cpre and Cpost represent the insulin or glucagon concentration before and after circulation, and Htpre and Htpost represent the hematocrit value before and after circulation.

### 4.2. Clinical Study

The study protocol was approved by the institutional ethics committee of Nihon University Itabashi Hospital (Approval No. RK-220614-2). All procedures were performed in accordance with the Helsinki Declaration of 1964 and its later amendments and adhered to national regulations. The study was registered in the University Hospital Medical Information Network (Registration No. UMIN000049856). Twenty patients with type 2 diabetes and end-stage kidney disease who were receiving tri-weekly HD on a regular basis provided written informed consent to participate in the study. Each patient received dialysis on two separate occasions with the two different dialyzers in a random order.

Each HD treatment session lasted 4 h (blood flow rate, 200 mL/min; dialysate flow rate, 500 mL/min; dialysate temperature, 37.0 °C). The same two types of dialyzers used in the experimental study (CTA or PS membrane) were used for each patient. The glucose concentration of the dialysate was 100 mg/dL. For anticoagulation, 2600–4000 units of standard heparin (Nipro Co., Ltd., Osaka, Japan) was administered per HD session. All parameters, including the clinical dry weight and drug prescription (insulin injection dosage and antidiabetic agents), were unchanged during the follow-up period. Patients were not permitted to eat or receive glucose solution injections during the HD sessions [5].

Blood samples were obtained from site A at the beginning and end of the HD session to evaluate the reduction rates of plasma insulin, glucagon, β2MG, and α1MG. One hour after starting dialysis, blood samples were obtained from sites A and V to evaluate the clearance of serum UN, Cr, β2MG, insulin, and glucagon. The ultrafiltration flow was stopped for 5 min before the blood samples were collected.

Serum insulin levels were analyzed using a chemiluminescent enzyme immunoassay (Chemilumi Insulin, Siemens Healthineers Japan, Tokyo, Japan), with a detection limit of 0.30 μIU/mL. Plasma glucagon levels were analyzed using sandwich enzyme-linked immunosorbent assay (Glucagon ELISA, Cosmic Corporation, Tokyo, Japan), with a detection limit of 3.5 pg/mL. Laboratory values such as serum UN, Cr, and plasma glucose were measured in our clinical laboratory using a biochemical autoanalyzer.

### 4.3. Statistical Analysis

Data are reported as the number and proportion, mean ± standard error, or median (interquartile range) as appropriate. Intragroup comparisons were made using two-tailed paired *t*-tests. Categorical variables were examined using the chi-squared test, and continuous variables were examined using the *t*-test. All analyses were performed using JMP^®^ version 13.0 (SAS Institute Inc., Cary, NC, USA). Statistical significance was set at *p*-values less than 0.05.

## Figures and Tables

**Table 1:** Concentration of insulin and glucagon at the arterial (A), venous (V), and ultrafiltrate fluid (F) sites.

**a**							
	**Dialyzers**	**5 min, QB = 200 mL/min, QF = 15 mL/min**			**5 min, QB = 200 mL/min, QF = 30 mL/min**		
		**A**	**V**	**F**	**A**	**V**	**F**
Insulin (μIU/mL)	CTA	6010 ± 1767	5703 ± 1701	3273 ± 1105	5830 ± 1769	5640 ± 1720	5230 ± 1382
	PS	5013 ± 1142	854 ± 219	0	5120 ± 1107	712 ± 180	0
Glucagon (pg/mL)	CTA	164,667 ± 3929	118,667 ± 9024	35,133 ± 3766	148,000 ± 6658	136,333 ± 7688	86,566 ± 19,766
	PS	82,667 ± 2333	29,633 ± 1449	1540 ± 329	54,367 ± 8888	25,333 ± 2669	588 ± 43
**b**							
	**Dialyzers**	**60 min, QB = 200 mL/min, QF = 15 mL/min**			**60 min, QB = 200 mL/min, QF = 30 mL/min**		
		**A**	**V**	**F**	**A**	**V**	**F**
Insulin (μIU/mL)	CTA	5806 ± 1731	5596 ± 1640	5536 ± 1616	5810 ± 1667	5696 ± 1664	5163 ± 1477
	PS	47.6 ± 7.6	29.9 ± 6.1	0	41.0 ± 6.7	27.2 ± 4.3	0
Glucagon (pg/mL)	CTA	139,333 ± 8950	119,000 ± 7371	111,500 ± 6331	137,667 ± 881	128,333 ± 1201	112,833 ± 12,657
	PS	2447 ± 160	2140 ± 113	857 ± 21	2347 ± 173	2190 ± 146	760 ± 24

a, At 5 min after starting circulation; b, at 60 min after starting circulation. A, Arterial site; CTA, cellulose-triacetate; F, filtration fluids; PS, polysulfone; QB, blood flow rate; QF, ultrafiltration rate; V, venous site.

**Table 2:** Patient characteristics in the clinical study.

*n* (male/female)	20 (13/7)
Age, years	70.8 ± 1.1
Body mass index, kg/m^2^	22.7 ± 0.4
Dialysis duration, months	46 (37–69)
History of CVD, *n* (%)	5 (25.0)
Primary disease of CKD, *n* (%)	
Diabetic nephropathy	16 (80.0)
Chronic glomerulonephritis	2 (10.0)
Nephrosclerosis	1 (5.0)
Other	1 (5.0)
Systolic blood pressure, mmHg	144 ± 2
Diastolic blood pressure, mmHg	80 ± 2
Heart rate, bpm	74 ± 2
Hemoglobin, g/dL	11.0 ± 0.1
Albumin, g/dL	3.8 ± 0.1
Serum urea nitrogen, mg/dL	63 ± 3
Creatinine, mg/dL	8.8 ± 0.6
Uric acid, mg/dL	6.9 ± 0.2
Calcium, mg/dL	8.7 ± 0.1
Phosphate, mg/dL	5.6 ± 0.1
Casual plasma glucose, mg/dL	159 ± 5
HbA1c, %	6.0 ± 0.1
Glycated albumin, %	20.3 ± 0.3
Medication, *n* (%)	
Insulin therapy	6 (30.0)
DPP-4 inhibitors	16 (80.0)
GLP-1 receptor agonists	1 (5.0)
α-glucosidase inhibitors	3 (15.0)
Diet and exercise therapy alone	2 (10.0)

CKD, Chronic kidney disease; CVD, cardiovascular disease; DPP-4, dipeptidyl peptidase-4; GLP-1, glucagon-like peptide-1; HbA1c, glycated hemoglobin.

## Data Availability

Data that support the findings of this study are available from the corresponding author upon reasonable request.

## Supplementary Materials

The following supporting information can be downloaded at: https://www.mdpi.com/article/10.3390/ijms241310604/s1.
