# Supplementary material for: Kinetics of Glucoregulatory Peptide Hormones during Hemodialysis with Cellulose Triacetate and Polysulfone Dialyzers in Patients with Diabetes and End-Stage Kidney Disease
Source: Int J Mol Sci. 2023 Jun 25;24(13):10604. doi: 10.3390/ijms241310604 (PMC10341656; doi:10.3390/ijms241310604)
Supplement: Supplementary file 1 [file ijms-24-10604-s001.zip › ijms-2435767-supplementary.pdf]

Supplementary Table S1. Technical specifications of the two super high-flux dialyzer membranes used in the study

* Dialyzer	FB-150Uβeco	APS-15SA
Membrane material	Cellulose triacetate	Polysulfone
Surface area (m^2)	1.5	1.5
Inner diameter (μm)	200	185
Wall thickness (μm)	15	45
Blood side volume (mL)	90	82
UFR (mL/mm Hg/h)	44	63

*Manufacturers: FB-150Uβeco, Nipro, Osaka, Japan; APS-15SA, Asahi Kasei Medical, Tokyo, Japan. UFR, ultrafiltration rate.
